# Immunopharmacological Properties of Methacrylic Acid Polymers as Potential Polymeric Carrier Constituents of Anticancer Drugs

**DOI:** 10.3390/molecules26164855

**Published:** 2021-08-11

**Authors:** Olga V. Zhukova, Evgenia V. Arkhipova, Tatyana F. Kovaleva, Sergey A. Ryabov, Irina. P. Ivanova, Anna A. Golovacheva, Daria A. Zykova, Sergey D. Zaitsev

**Affiliations:** 1 Federal State Budgetary Educational Institution of Higher Education, Privolzhsky Research Medical University of the Ministry of Health of the Russian Federation, 603950 Nizhny Novgorod, Russia; arhipova@nnovgorod.ru (E.V.A.); prazina@yandex.ru (T.F.K.); zykovazda@gmail.com (D.A.Z.); 2 Department of High-Molecular Compounds and Colloid Chemistry, National Research Lobachevsky State University, 603022 Nizhny Novgorod, Russia; ryabov_sa@mail.ru (S.A.R.); ivanova.ip@mail.ru (I.P.I.); a.golovachiova@yandex.ru (A.A.G.); szay@inbox.ru (S.D.Z.)

**Keywords:** (co)polymers of methacrylic acid, molecular weight characteristics of (co)polymers, immune system, interleukins, cytokines

## Abstract

Cytostatic chemotherapeutics provide a classical means to treat cancer, but conventional treatments have not increased in efficacy in the past years, warranting a search for new approaches to therapy. The aim of the study was, therefore, to obtain methacrylic acid (MAA) (co)polymers and to study their immunopharmacological properties. 4-Cyano-4-[(dodecylsulfanylthiocarbonyl)sulfanyl] pentanoic acid (CDSPA) and 2-cyano-2-propyl dodecyl trithiocarbonate (CPDT) were used as reversible chain transfer agents. Experiments were carried out in Wistar rats. The MTT assay was used to evaluate the cytotoxic effect of the polymeric systems on peritoneal macrophages. An experimental tumor model was obtained by grafting RMK-1 breast cancer cells. Serum cytokine levels of tumor-bearing rats were analyzed. The chain transfer agents employed in classical radical polymerization substantially reduced the molecular weight of the resulting polymers, but a narrow molecular weight distribution was achieved only with CDSPA and high CPDT concentrations. Toxicity was not observed when incubating peritoneal macrophages with polymeric systems. In tumor-bearing rats, the IL-10 concentration was 1.7 times higher and the IL-17 concentration was less than half that of intact rats. Polymeric systems decreased the IL-10 concentration and normalized the IL-17 concentration in tumor-bearing rats. The maximum effect was observed for a MAA homopolymer with a high molecular weight. The anion-active polymers proposed as carrier constituents are promising for further studies and designs of carrier constituents of drug derivatives.

## 1. Introduction

Cytostatic chemotherapeutics that target nucleic acids and signaling pathways regulating cell proliferation provide a classical means to treat cancer [1,2]. Conventional treatments (surgery, chemotherapy, and radiotherapy) have not increased in efficacy in the past years, warranting a search for new approaches to therapy [3,4]. Polymeric carrier constituents are promising as vehicles for drug delivery because they increase the solubility of hydrophobic agents, prolong the drug life in circulation, and may improve the biodistribution profile of a low-molecular-weight drug [5].

Various forms of polymeric carrier constituents have been developed and investigated throughout the world to deliver anticancer drugs [6]. For example, polymeric nanogels, which are capable of a reversible response to minor changes in external factors (temperature, ion strength, pH, electrical field, etc.) and may incorporate and release an active drug substance in a controlled manner, and amphiphilic polymeric micelles, which can harbor a drug in their core [7]. Systems based on polyethylene glycol, polylactides, and polyglutamic acid have been described in the literature, and polyacrylic acid has been noted as a potential drug carrier [8]. Polymeric systems are tested for cytotoxicity and antitumor efficacy in vivo in the course of their development [9]. However, it is still poorly understood how the structures and molecular weight characteristics of polymeric carrier constituents are associated with their biopharmaceutical properties. 

An important current problem is the design of anticancer drug delivery systems that enhance, rather than just retain, the cytotoxic effect of a drug on tumor tissue and simultaneously reduce its toxic effect on other organs, as well as the design of the means to activate the immune antitumor mechanisms [10,11,12,13]. Polymethacrylic acid combined with gold nanoparticles and joined with doxorubicin through an acid-labile cysteine bond was tested for anticancer properties in vitro and in vivo. A high efficacy of the conjugate was demonstrated with a human cervical adenocarcinoma cell line in both chemotherapy and radiotherapy [14]. A copolymer of polymethacrylic acid and polyethylene glycol was synthesized and used as a coating of magnetic nanoparticles to deliver cisplatin, which is a potent anticancer agent with a nonselective effect. Polymer-based cisplatin derivatives displayed higher anticancer activity and lower toxicity as compared with pure cisplatin. However, the study did not detect a substantial increase in the cisplatin concentration in the tumor site. The finding indicates that the enhanced anticancer effect was due to mechanisms other than higher cisplatin accumulation in the tumor [15]. Combinations of anticancer and immunomodulatory agents are often used to treat cancer. They improve the efficacy of therapy, and the immunomodulatory component often possesses anti-inflammatory activity [16,17,18].

The mechanisms that activate anticancer immunity include a regulation or stimulation of each step in cancer–immunity interactions and are aimed, in particular, at increasing the amounts and functional activities of cytotoxic T cells and natural killers, the production of antibodies in B cells, and cell secretion of cytokines, factors stimulating immune cell proliferation, or factors suppressing the growth of tumor cell clones [19,20,21].

Polymeric particles are of particular interest in this respect when used as anticancer drug carrier constituents [22]. Synthetic polyelectrolytes are not antigenic. However, they are known to enhance the immune response when administered together with antigens, acting as adjuvants. Advantage of this property has been taken in designing vaccines [23,24,25]. Immunostimulatory activity of polymeric adjuvants is based on their macromolecular nature. One of the relevant properties is that polymeric adjuvants are capable of cooperative interactions with chemically complementary molecules to produce stable interpolymer complexes or tight multipoint cooperative adsorption on chemically complementary surfaces [26,27]. The immune response starts with recognition of alien antigens and results in an accumulation of effector immune cells and antibodies. B cells, T cells, and macrophages are the main cells involved in the immune response. Linear polyelectrolytes can mediate adhesion between B and T helper cells via their multipoint adsorption on the cell membranes. Synthetic polyelectrolytes increase the effect of T–B cell cooperation [28]. Immunostimulatory and anticancer activity of polyanions is due to their direct effect and the ability to activation macrophages [29]. Activated macrophages selectively ingest cancer cells, in contrast to nonactivated macrophages. The role that macrophages play in carcinogenesis has been considered in many works, including systematic reviews [30,31,32].

Tumor-associated macrophages (TAMs) have received special attention over the past 30 years, starting from the formulation of the macrophage dichotomy concept [33,34]. TAMs are classed as type II-activated macrophages (M2). Stein et al. (1992) have initially characterized TAMs as alternatively activated macrophages [35,36]. Data on TAM markers and TAM-suppressing factors have accumulated in further research. The M2 population is highly heterogeneous [37,38]. Macrophages of the M2 phenotype play an important role in the development of the tumor process by suppressing the immune response, remodeling the extracellular matrix, and stimulating angiogenesis [35]. Macrophages of the M1 phenotype (classically activated macrophages) are characterized by expression of bactericidal molecules and receptors [39]. Macrophages acquire the M1 phenotype in response to endogenous inflammatory stimuli, such as the Th1-asscoiated cytokine interferon gamma, or exogenous inflammatory stimuli, such as lipopolysaccharide [40]. M1 macrophages facilitate tumor cell elimination, while M2 macrophages promote carcinogenesis [41].

Expression of inhibitory cytokines by tumor cells or macrophages is one of the mechanisms responsible for resistance to anticancer therapy. A therapeutic strategy that targets macrophages or macrophage-derived cytokines may hold promise for effective cancer treatment [42]. Increasing the efficacy of anticancer therapy, expanding the therapeutic ranges of current drugs, improving the selectivity of their effects, overcoming multiple drug resistance, and stimulating the immune system to more efficiently fight cancer in tumor bearers are pressing problems of modern science and medicine.

Based on these premises, the aim of the study was to obtain methacrylic acid (MAA) (co)polymeris and to study their immunopharmacological effects. Anion-active polymers proposed as carrier constituents normalize the cytokine levels and are, therefore, promising for the further study and design of drug derivatives.

## 2. Results

### 2.1. Synthesis of Polymers and Their Molecular Weight Characteristics

Two approaches were used to obtain MAA-based polymers. One was direct MAA polymerization in a dioxane solution to ensure homogeneous polymerization, and the other included tert-Butyl methacrylate (TBMA) polymerization and subsequent modification (acid hydrolysis) of ester groups in the polymer. All polymers were synthesized via reversible addition–fragmentation chain transfer polymerization (RAFT). Molecular weight characteristics of polyMAA obtained in the presence of CDSPA and CPDT are summarized in Table 1. The RAFT agents substantially reduced the molecular weight of polymers synthesized by classical radical polymerization and narrowed their molecular weight distributions, but a narrow molecular weight distribution was achieved only with CDSPA and high CPDT concentrations. As is well known, pseudoliving RAFT (co)polymerization of unsaturated carboxylic acids is difficult to achieve. [43] We therefore used RAFT polymerization of TBMA with subsequent acid hydrolysis of ester groups in the polymer to obtain MAA-based polymers. 

As is seen from Table 2, a narrow molecular weight distribution was observed for the polymers synthesized in the presence of CPDT throughout the RAFT concentration range.

When polyTBMA polymers were subjected to acid hydrolysis, the extent of hydrolysis was 77.87 ± 1.65%.

The cytotoxic effect of synthetic polymers on immune system cells (peritoneal macrophages) was tested using polyMAA (system 1: M_n_∙10^3^ = 16.0, M_w_/M_n_ = 1.47; system 2: M_n_∙10^3^ = 99.2, M_w_/M_n_ = 1.57; and system 3: M_n_∙10^3^ = 19.0, M_w_/M_n_ = 1.45) and TBMA–MAA copolymers produced via hydrolysis (system 4: M_n_∙10^3^ = 21.5, M_w_/M_n_ = 1.33; system 5: M_n_∙10^3^ = 13.8, M_w_/M_n_ = 1.13). 

**Figure 1:**
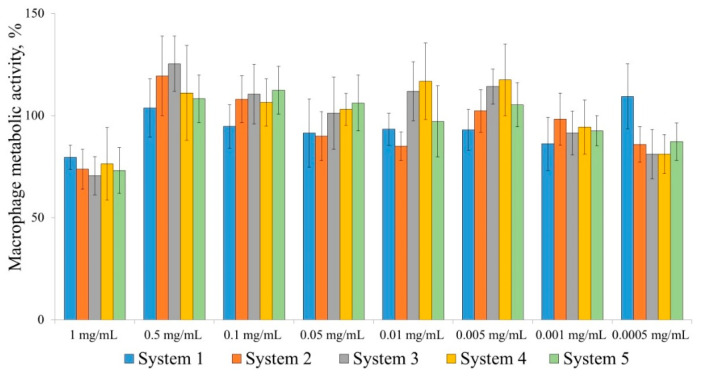
Metabolic activity measured in the MTT assay for peritoneal macrophages incubated with the polymers for 4 h. Data presented are the mean ± SD of eight independent experiments each performed in triplicate. System 1, polyMAA (M_n_∙10^3^ = 16.0 Da, M_w_/M_n_ = 1.47); system 2, polyMAA (M_n_∙10^3^ = 99.2 Da, M_w_/M_n_ = 1.57); system 3, polyMAA (M_n_∙10^3^ = 19.0 Da, M_w_/M_n_ = 1.45); system 4, TBMA–MAA copolymer (M_n_∙10^3^ = 21.5 Da, M_w_/M_n_ = 1.33); system 5, TBMA–MAA copolymer (M_n_∙10^3^ = 13.8 Da, M_w_/M_n_ = 1.13).

Because the synthetic polymers are intended for use as potential anticancer drug carrier constituents, they will interact with the immune system in circulation. To study the immune cell response to the (co)polymers, we determined their concentrations at which 50% of cells lost their viability (IC50). Peritoneal macrophages were incubated with various polymers at their various concentrations for 4 h, and all of the systems tested were found to be nontoxic when used at 1 mg/mL (Figure 1). 

When peritoneal macrophages were incubated with various polymers at their various concentrations for 24 h, all of the systems tested were nontoxic at 0.5 mg/mL, which is a high concentration (Table 3). 

### 2.2. Effects of the MAA Polymers on the Cytokine Levels during Tumor Development

Immunostimulatory activity of the synthetic MAA (co)polymers were evaluated in vivo, using a RMK-1 breast cancer cell graft model in rats. 

The polymeric carrier constituents were tested for effect on cytokine production in rats with RMK-1 tumors. Experiments were carried out with system 5, which was a TBMA–MAA copolymer and had the lowest molecular weight and the narrowest molecular weight distribution, and system 2, which was a MAA homopolymer with a relatively high molecular weight (99 kDa). The aim was to evaluate the effect of the molecular weight on immune system parameters. 

The MAA (co)polymers were administered on day 10 of tumor development. The results showed that the IL-10 and IL-17 concentration tended to change (normalize) in rats. 

**Figure 2:**
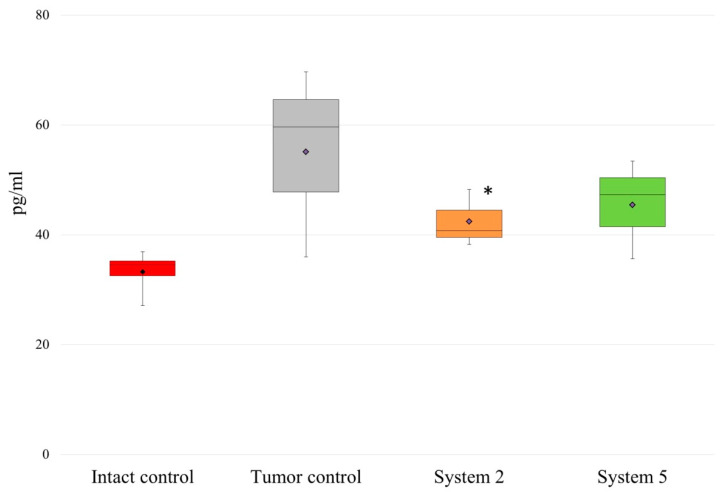
Changes in IL-10 concentration in response to administration of the polymeric systems. Rats were administered i.p. with 9 mg/kg polymeric system (System 2 or System 5) once on the 10th day, which corresponded to the beginning of oncogenesis and formation of tumor nodes, and the activation of the immune system. Control rats received PBS. Results are representative of four independent assays and show the Median (Me) with interquartile range (25th percentile; 75th percentile) of triplicate determinations for one of four experiments. Differences from (*) intact rats were significant at *p* < 0.05.

On day 10 of tumor development, the serum IL-10 concentration increased in tumor-bearing rats by a factor of 1.7, to 55.00 ± 17.30 pg/mL, compared with 33.26 ± 4.27 pg/mL in intact rats (control). Administration of the polymeric systems decreased the IL-10 concentration in the tumor-bearing rats to 45.45 ± 9.02 pg/mL in the case of system 2 and 42.40 ± 5.21 pg/mL in the case of system 5 (Figure 2).

**Figure 3:**
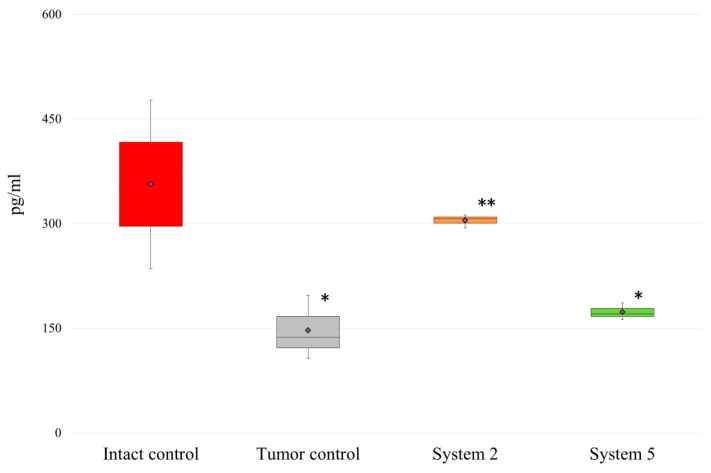
Changes in IL-17 concentration in response to administration of the polymeric systems. Rats were administered i.p. with 9 mg/kg polymeric system (System 2 or System 5) once on the 10th day, which corresponded to the beginning of oncogenesis and formation of tumor nodes, and the activation of the immune system. Control rats received PBS. Results are representative of four independent assays and show the Median (Me) with interquartile range (25th percentile; 75th percentile) of triplicate determinations for one of four experiments. Differences from (*) intact rats or (**) control tumor-bearing rats were significant at *p* < 0.05.

The IL-17 concentration decreased more than twice in tumor-bearing rats compared with intact rats (147.13 ± 45.96 pg/mL vs. 356.53 ± 120.58 pg/mL, respectively) and was normalized after administration of the polymeric systems. Normalization was more efficient in the case of the MAA homopolymer with a high molecular weight (Figure 3).

## 3. Discussion

RAFT polymerization was used to obtain MAA (co)polymers with controlled molecular weight characteristics. The method has several apparent advantages over other radical processes with reversible chain deactivation. For example, atom transfer radical polymerization (ATRP) inevitably leads to undesirable contamination of the polymer with transition metal complexes, which are used as catalysts, and stable free radical polymerization (SFRP) often requires a temperature higher than 100 °C. Among all pseudoliving radical processes, RAFT has apparent advantages of being efficient, simple, and universal, being compatible with virtually all monomers that are capable of radical polymerization, and allowing efficient control over the molecular weight characteristics of the resulting polymers. Moreover, RAFT offers great opportunities for macromolecular design and makes it possible to obtain specific materials with diverse functional potentials, such as block, graft, star-like, comb-like, and gradient copolymers.

Radical polymerization was studied for TBMA and MAA in the presence of the RAFT agents CPDT and CDSPA. Polymerization was shown to follow a pseudoliving mechanism, which is evident from the fact that the number average molecular weight increases with increasing conversion. Low dispersity was observed for the resulting polymers, together with lack of a gel effect. 

The synthetic MAA (co)polymers, which are proposed as drug carrier constituents, were found to exert no toxic effect on immune system cells when incubated with macrophages at 1 mg/mL for 4 or 0.5 mg/mL for 24 h. The (co)polymers did not change the metabolic activity of macrophages and are thus promising for the further investigation and construction of drug derivatives. 

It should be noted that PMAA itself has antitumor effects [44]. PMAA is known as a potential carrier of antitumor drugs. In 2016, a study was carried out to study the antitumor properties in vitro and in vivo of PMAA, combined with gold-containing nanoparticles, as well as with doxorubicin through an acid-labile cysteine bond [45]. On the cell line of human cervical adenocarcinoma the high efficiency of the conjugate both in chemotherapy and radiation therapy has been demonstrated.

However, there was no assessment of the independent influence of PMAA on the tumor process, or on the state of immune system in the development of the tumor process. This fact defines the prospects of the presented research.

Cytokines are an important element of the immune system. Cytokines regulate the intercellular and intersystemic interactions that determine the cell viability, stimulate or suppress the cell growth, differentiation, functional activity, and apoptosis, and ensure the concerted functions of the immune, endocrine, and nervous systems in normal conditions and in response to pathological factors [46]. 

The IL-10 and IL-17 levels tended to change in response to administration of MAA (co)polymers. Both of the cytokines are known to play a dual role in the tumor process according to published data [47,48,49].

The pro-oncogenic effects most commonly mentioned for IL-10 are that IL-10 reduces the antitumor immune response in the tumor microenvironment, thus helping tumor cells to evade the effects of immune cells and stimulates angiogenesis [50,51,52]. Stimulation of natural killer cells and inhibition of reactive oxygen species are noteworthy among the antitumor effects of the cytokine [52].

Activation of angiogenesis is a prooncogenic effect of IL-17. Its antitumor effects include stimulation of the antitumor cytotoxic T-cell response [53].

**Figure 4:**
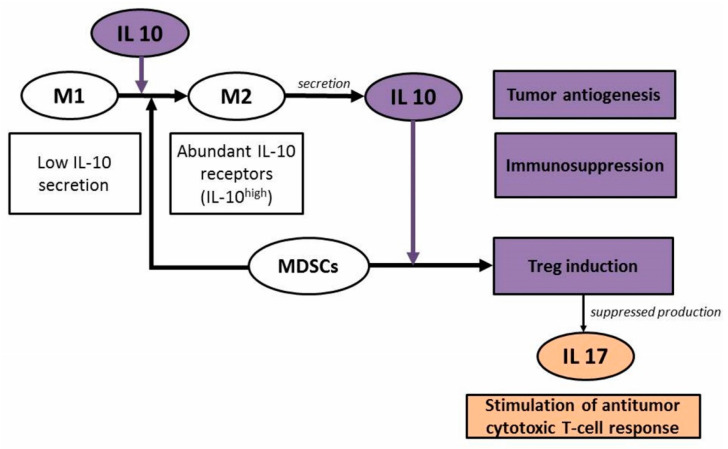
Roles that IL-10 and IL-17 play in the tumor process. MDSCs: myeloid-derived suppressor cells. Treg (regulatory T cells: T-regulatory cells, T suppressors) are central regulators of the immune response.

Summarizing the literature data, the following scheme might be assumed for the effects of IL-10 and IL-17 on the tumor process. IL-10 promotes conversion of M1 macrophages (classically activated, with phagocytosis as a main function) to M2 macrophages (alternatively activated macrophages, TAMs, which facilitate tumor cell evasion from the immune system) [44]. Moreover, M2 macrophages are known to express ample IL-10 receptors and to secrete IL-10 [53]. As a proinflammatory cytokine, IL-10 stimulates the suppressor cells, the main function of which is to inhibit secretion of cytokines, including IL-17. Stimulation of the antitumor cytotoxic T-cell response is thus suppressed, and the tumor process spreads [48] (Figure 4).

## 4. Materials and Methods

### 4.1. Chemicals and Reagents

Tert-Butyl methacrylate (TBMA) and MAA monomers (Sigma-Aldrich, Munich, Germany) were used to synthesize polymers. TBMA and MAA were purified by distillation under reduced pressure before use. The initiator azoisobutyric acid dinitrile (AIBN) (Sigma-Aldrich, Munich, Germany) was recrystallized from methyl-tert-butyl ester, dried under reduced pressure, and stored at 0 °C. The purity was checked by ^1^H NVR spectroscopy. ^1^H NMR (CDCl_3_, δ, ppm): 1.73 (s, 11H). 4-Cyano-4-[(dodecylsulfanylthiocarbonyl)sulfanyl] pentanoic acid (CDSPA) (97%, TCI, Tokyo, Japan) and 2-cyano-2-propyl dodecyl trithiocarbonate (CPDT) (Sigma-Aldrich, Munich, Germany) were used as reversible addition–fragmentation chain transfer (RAFT) agents. The latter was synthesized according to a published protocol [54]. The solvents dioxane, methanol, tetrahydrofuran (THF), and dimethyl sulfoxide were purified by conventional methods [55].

The RAFT-prepared polyMAA polymers were characterized by ^1^H NMR techniques. In cases in which the CDSPA terminated polymer ^1^H NMR (400 MHz, DMSO-d6) spectra shows resonance peaks at δ (ppm): 3.6 (s, ‒COOH), 3.3 (m, ‒CH_2_‒COOH), 2.6 (m, ‒CH_2_‒CH_2_‒COOH), 2.4-2.5 (tr, ‒CH_2_‒S‒), 1.7 (m, ‒CH_2_‒CH_2_‒S‒), 1.8 (s, ‒C(CH_3_)CN‒), 1.3 (s, ‒(CH_2_)_10_‒), 0.8 (tr, ‒CH_3_). In cases in which the CPDT terminated polymer ^1^H NMR (400 MHz, DMSO-d6) spectra shows resonance peaks at δ (ppm): 3.3 (tr, ‒CH_2_‒S‒), 1.7 (m, ‒CH_2_‒CH_2_‒S‒), 1.9 (s, ‒C(CH_3_)_2_CN), 1.3 (s, ‒(CH_2_)_10_‒), 0.8 (tr, ‒CH_3_).

### 4.2. Polymerization

Batch polymerization of TBMA was carried out in the presence of CPDT. MAA was polymerized in a dioxane solution (monomer: solvent = 2:1 *v*/*v*) in the presence of CPDT and CDSPA at 70 °C. Polymerization was carried out in ampoules sealed after a preliminary degassing via three rounds of freezing–thawing under vacuum. The polymerization durations were 6 h in the case of TBMA and 8 h in the case of MAA. The initiator concentration relative to the monomer was 2·10^−3^ mol/L. The RCT agent concentration varied from 0.01 to 0.1 mol/L. The resulting polymers were purified via precipitation with chilled methanol from a chloroform solution (in the case of polyTBMA) or chilled diethyl ester from an ethanol solution (in the case of polyMAA); the purification procedure was repeated three times. The polymers were dried under vacuum to a constant weight at room temperature. 

In addition to the elementary reactions of classical radical polymerization (chain initiation, growth, and termination), RAFT includes reversible chain transfer to a sulfur compound, Z−C(=S)−S−R, where Z is the stabilizing group and R is the leaving group, which is easily cleaved via a radical mechanism (reactions 1–2):
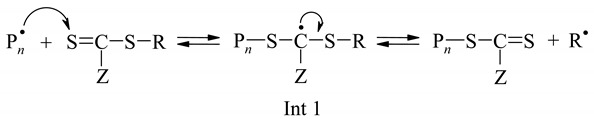
(1)

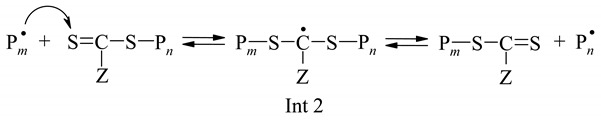
(2)

Fragmentation of an intermediate formed in the last reaction yields new microradicals, which are involved in the chain growth reaction until the next addition of the RAFT agent to the polymer, leading to a conversion increase in polymer molecular weight and a narrower molecular weight distribution.

### 4.3. Molecular Weight Characteristics of Polymers

Molecular weight characteristics of polyMAA were studied by gel permeation chromatography (GPC); carboxyl groups were preliminarily methylated with diazomethane. GPC was carried out on a Shimadzu Prominence LC–20VP system with Tosoh Bioscience columns packed with polystyrene gel with pore sizes of 1 × 10^5^ and 1 × 10^4^ Å, using THF as an eluent at a flow rate of 0.7 mL/min at 40 °C. A differential refractometer was used as a detector. Chromatograms were analyzed using LCsolution software. Narrow-disperse PMMA standards were used for calibration. 

### 4.4. PolyTBMA Hydrolysis

PolyTBMA was hydrolyzed in dioxane supplemented with diluted (1:2) HCl at 2 mL of the solvent per 1 g polymer at 100 °C, using a flask fitted with a reflux condenser. After polyTBMA hydrolysis, the MAA unit content in the copolymer was determined by potentiometric titration of acidic groups in methanol with 0.1 N KOH. 

### 4.5. Experimental Animals

Experiments were carried out in Wistar rats (females; weight: 260.0 ± 10 g; age at the beginning of the experiment: 3 months). Animal rearing at the certified breeding facility of the Central Research Laboratory of the Privolzhsky Research Medical University (RF Ministry of Health) complied with the Health and Hygiene Standards SP 2.2.1.3218-14. All experiments were performed in accord with the Guide for the Care and Use of Laboratory Animals (National Research Council, 2011) and the European Convention for the Protection of Vertebrate Animals Used for Experimental and Other Scientific Purposes (Strasbourg, 2006) and were approved by the Ethics Committee at the Privolzhsky Research Medical University.

### 4.6. Primary Peritoneal Macrophage Cultures

To obtain a macrophage suspension, 20 mL of sterile physiological solution was injected into rats intraperitoneally. After 20 min, the animals were decapitated under isoflurane anesthesia. Peritoneal fluid was collected with sterile pipette in tube, centrifuged and diluted to working concentration. A cell suspension (2 × 10^6^ cells/mL) in RPMI-1640 (Biosera, Nuaille. France) supplemented with 10% fetal bovine serum (Biosera, Nuaille, rance) was transferred into culture plates for cell visualization and analysis. The plates were incubated in a CO_2_ incubator for 60 min to allow cell adhesion. A macrophage monolayer was washed twice with phosphate-buffered saline to remove nonadherent cells and incubated in an atmosphere containing 5% CO_2_ at 37 °C for 1 h. The medium was changed, test polymers were added to various concentrations (5 × 10^−4^ mg/mL, 1 × 10^−3^ mg/mL, 5 × 10^−3^ mg/mL, 1 × 10^−2^ mg/mL, 5 × 10^−2^ mg/mL, 0.1 mg/mL, 0.5 mg/mL, 1 mg/mL) to determine the IC_50_, and the cultures were incubated for 4 or 24 h. 

### 4.7. Cytotoxicity of Polymers to Immune System Cells (Peritoneal Macrophages) In Vitro; MTT Analysis

Cytotoxicity of polymeric systems to peritoneal macrophages was evaluated in the MTT assay [56]. The assay takes advantage of the fact that mitochondrial dehydrogenases of viable cells are capable of reducing the tetrazolium dye 3-(4,5-dimethylthiazol-2-yl)-2,5-diphenyltetrazolim bromide (MTT) to formazan, which forms crystals within cells. The purple color intensity (optical density) at 595 nm was measured on a plate spectrophotometer. The MTT assay results were evaluated by comparing the optical density between test and control wells. The optical density is proportional to the viable cell count in a well. Cytotoxic activity of an agent was inferred from the changes in optical density. A difference in optical density from a control was tested for significance using the Mann–Whitney test. A difference that was significant at *p* < 0.05 suggested a negative effect on cell viability for the test polymeric system. Saline (0.9% sodium chloride) was used as a control. 

### 4.8. The ExperimentalModel of Cancer In Vivo

The experimental model of neoplasia was obtained by transplantation of the tumor strain of breast cancer cells RMK-1 obtained from Blokhin Cancer Research Center. Transplantation began with anesthesia of the donor rat, then the subcutaneous tumor was cut out and crushed, as a result of which cancer cells were suspended in sterile Hanks’ solution at a ratio of 50 mg per 0.5 mL. Cell suspension was injected to the recipient rat subcutaneously into the armpit area axillary region. The day of transfusion of injection was taken as 0 days of tumor development. Test compounds were injected intraperitoneally with 9 mg/kg polymeric system (System 2 and System 5) once on the 10th day, which corresponded to the beginning of oncogenesis and formation of tumor nodes and activation of the immune system [57]. Control rats received PBS. On the 16th day, animals were decapitated under isoflurane anesthesia. The tumor size was measured, using a caliper on the 16th day; the volume was calculated as: V = a  ∙  b ∙  b/2, where a—length of tumor; b—width of tumor.

To assess the level of cytokines, blood was collected in a test tube, centrifuged and serum was taken away. Suspension of peritoneal macrophage cultures was obtained in the same way as for primary peritoneal macrophage cultures. 

To assess the effect of methacrylic acid polymers on the immune system, the animals were divided into the following groups: healthy animals without tumors (Intact control) in the amount of 4; animals with RMK-1 (Tumor control) in the amount of 4; animals with RMK-1, which were administered system 2 (System 2), in the amount of 4; animals with RMK-1, which were administered system 5 (System 5), in the amount of 4.

### 4.9. Serum Cytokine Level Measurements in Tumor-Bearing Rats by Flow Cytometry

IL-10 and IL-17 were measured in the blood serum by ELISA, using Cloud-Clone kits (United States) and an Epoch spectrophotometer (BioTek, United States).

### 4.10. Statistical Analysis

The mean values (M) and standard deviations (SD) were calculated to express the data. Quantitative variables were described by median (Me) with interquartile range (25th percentile; 75th percentile) in the case of a non-normal distribution or the mean (M) and standard deviation (SD) if the distribution was normal. The Mann–Whitney test was used to assess the significance of differences between the two groups (*p* < 0.05 was considered statistically significant).

## 5. Conclusions

The anion-active polymers that we propose as carrier constituents normalize the levels of cytokines (IL-10, IL-17) and are, therefore, promising for the further research and design of drug derivatives. 

Polymers with high molecular weights and charges are capable of activating the immune system and, in particular, the macrophage system, thus exerting an indirect antitumor effect. Polymers that possess intrinsic immunostimulatory activity may help to eliminate the side effects that chemotherapy exerts on the immune system. The role that the immune system plays in tumorigenesis and tumor suppression is currently receiving particular attention. 

A transition from the small molecules currently used as anticancer drugs to polymeric systems makes it possible to combine the drugs with polymeric carrier constituents possessing intrinsic pharmacological activity. This may help to optimize anticancer therapy in medicine by increasing the specificity of drug interactions with cancer cells, reducing the side effects, and thus expanding the therapeutic ranges of drugs.

## Figures and Tables

**Table 1:** Molecular weight characteristics of polyMA polymers synthesized in the presence of CPDT and CDSPA, AIBN = 0.002 mol/L, T = 70 °C.

RAFT, mol/L	CPDT			CDSPA		
	M_n_∙10^3^	M_w_∙10^3^	M_w_/M_n_	M_n_∙10^3^	M_w_∙10^3^	M_w_/M_n_
0.01	87.8	149.8	1.71	99.2	160.4	1.57
0.04	31.3	45.9	1.47	31.5	40.0	1.27
0.08	19.0	27.7	1.45	19.5	23.6	1.19
0.10	16.0	22.7	1.47	14.6	16.6	1.13

**Table 2:** Molecular weight characteristics of polyTBMA synthesized in the presence of CPDT, AIBN = 0.002 mol/L, T = 70 °C.

RAFT, mol/L	CPDT		
	M_n_∙10^3^	M_w_∙10^3^	M_w_/M_n_
0.01	46.9	53.5	1.14
0.04	21.5	28.7	1.33
0.08	13.8	15.6	1.13
0.10	11.5	13.2	1.15

**Table 3:** IC50 (mg/mL) measured for the polymeric systems in 24 h incubation with macrophages.

Polymer	IC50, mg/mL
System 1	0.707
System 2	0.612
System 3	0.652
System 4	0.657
System 5	0.567

## Data Availability

The data presented in this study are available on request from the corresponding author.
